# The effect of blood flow restriction training with different intermittent durations on the post-activation potentiation of lower limbs:a meta-analysis

**DOI:** 10.3389/fphys.2026.1777491

**Published:** 2026-05-15

**Authors:** Xinrui Miao, Wenqing Chang, Yulin Yang

**Affiliations:** 1 Department of Physical Education, Kunsan National University, Gunsan, Republic of Korea; 2 College of Physical Education, Putian University, Putian Fujian, China; 3 School of Sports, Northeast Electric Power University, Jilin, Jilin, China

**Keywords:** back squat, blood flow restriction training, meta-analysis, post-activation potentiation enhancement, rest interval

## Abstract

**Objective:**

This meta-analysis aimed to systematically evaluate the effect of blood flow restriction (BFR) training with different intermittent durations on the post-activation potentiation (PAP) of lower limbs, and clarify the optimal rest interval window and related key training parameters.

**Methods:**

Randomized controlled trials (RCTs) investigating the impact of BFR training on lower limb PAP were retrieved from PubMed, Web of Science, EBSCO, and CNKI databases in accordance with the PRISMA statement. The search period covered from the establishment of each database to November 15, 2025. The Cochrane Risk of Bias Tool was used for literature quality assessment, Review Manager 5.3 software for statistical analysis, Stata 17.0 software for sensitivity analysis, and Egger test combined with funnel plots for detecting publication bias.

**Results:**

A total of 16 RCTs involving 281 participants were included. The meta-analysis showed that lower limb BFR training significantly induced PAP compared with the control group [*SMD* = 0.36, 95% *CI* (0.07, 0.65), P = 0.02]. Subgroup analysis indicated that rest interval, compressive strength, exercise mode, exercise intensity, and outcome extracted were potential sources of heterogeneity among studies. The largest effect sizes with statistical significance were observed in the following subgroups: rest interval (2~4 min) [*SMD* = 0.83, 95% CI (0.56, 1.11), *P* < 0.0001], compressive strength (40%~60% AOP) [*SMD* = 0.76, 95% *CI* (0.46, 1.06), *P* < 0.0001], exercise mode (Squat) [*SMD* = 0.49, 95% *CI* (0.02, 0.97), P = 0.04], exercise intensity (40%~70% 1RM) [*SMD* = 0.99, 95% *CI* (0.48, 1.51), *P* = 0.0002], and outcome extracted (Counter movement jump, CMJ) [*SMD* = 0.53, 95% *CI* (0.17, 0.89), *P* = 0.004]. Sensitivity analysis confirmed the stability of the results, and funnel plots combined with Egger test showed no significant publication bias (all *P* > 0.05).

**Conclusion:**

Lower limb BFR training can effectively induce PAP. For BFR interventions targeting lower limb PAP induction, the optimal potentiation effect is achieved with squat exercises at an exercise intensity of 40%~70% 1RM, a compressive strength of 40%~60% absolute occlusion pressure (AOP), and an inter-set rest interval of 2~4 min.

**Systematic review registration:**

https://www.crd.york.ac.uk/prospero/, identifier CRD42023478585.

## 1 Introduction

With the continuous improvement of competitive sports performance, traditional training methods have gradually shown limitations in breaking through the bottleneck of athletes’ competitive ability and further enhancing muscle explosive power. As a core determinant of athletic performance in explosive-dominant events such as jumping, throwing, and sprinting, the optimization and enhancement of muscle explosive power have long been a research focus and practical priority in the field of sports training (Maden-Wilkinson et al., 2021). To achieve this goal, athletes often adopt targeted intervention methods such as plyometric training and resistance training based on the characteristics of specific sports. In recent years, Blood Flow Restriction Training (BFR) has attracted extensive attention as an innovative training approach (Lorenz et al., 2021). Studies have confirmed that compared with traditional resistance exercises of the same intensity, BFR applies appropriate external pressure to the limbs to occlude distal venous blood flow, enabling more efficient improvements in muscle strength and hypertrophy even at low exercise intensities (Bielitzki et al., 2021; Li et al., 2023). Moreover, this training mode facilitates muscles to quickly reach an optimal functional state during the warm-up phase, laying a solid foundation for the enhancement of subsequent athletic performance (Frippiat and Votion, 2024).

Post-Activation Potentiation (PAP) refers to a physiological phenomenon in which the subsequent explosive power performance of muscles is temporarily improved after completing one set or a single bout of high-intensity, short-duration pre-contraction (Li et al., 2024). This effect provides an important physiological basis for optimizing athletic performance during training and competitions (Gallardo et al., 2024). However, controversies remain regarding the effective induction methods (e.g., load intensity, exercise type selection) and the optimal time window for the PAP effect (Georgogiannis and Tsalis, 2023). In practice, most coaches tend to adopt submaximal intensity resistance exercises as PAP induction methods, but such approaches are often accompanied by a certain degree of muscle fatigue accumulation, which may impair the practical application effect of the PAP effect (McKiel et al., 2024). In contrast, the low-intensity nature of BFR training gives it potential advantages in inducing the PAP effect—it can not only activate myogenic regulatory pathways through appropriate mechanical stimulation and metabolic stress but also reduce fatigue accumulation, creating favorable conditions for the full exertion of the PAP effect (Tian et al., 2022). Nevertheless, whether the exertion of this advantage depends on specific rest interval configurations has not been clearly verified.

Although relevant research on BFR training and the PAP effect has made certain progress, systematic integrated studies on the impact of different rest intervals—a key training variable—on the lower limb PAP effect remain relatively scarce (Celik and Erikoglu Orer, 2025). Existing empirical research results show obvious discrepancies: some studies suggest that short rest intervals (≤ 5 min) can preserve the metabolic stress advantages induced by BFR, which is more conducive to the expression of the PAP effect (Wang et al., 2023), other studies support that long rest intervals (≥ 10 min) can reduce fatigue interference and achieve better explosive power improvement (Wang et al., 2025). This result heterogeneity leads to a lack of unified evidence-based basis for coaches when formulating training programs, limiting the precise application of BFR training in optimizing the PAP effect. In view of this, this study intends to adopt a meta-analysis method to systematically retrieve and integrate relevant literature on the effect of BFR training on the lower limb PAP effect under different rest intervals, quantitatively analyze the correlation characteristics between rest intervals and the intensity and duration of the PAP effect, and clarify the optimal rest time window. The results of this study are expected to fill the methodological gaps in existing research, provide a scientific basis for optimizing training programs for PAP effect induction in athletes, and further enhance the application efficiency and practical value of BFR training in competitive sports.

## 2 Materials and methods

### 2.1 Search strategy

To enhance the transparency and credibility of the literature review process, we strictly followed the PRISMA statement (Schmidt et al., 2019) and implemented a detailed, double-blind search protocol conducted independently by two researchers. The search was performed across four core databases: PubMed, Web of Science, EBSCO, and CNKI, which were selected for their comprehensive coverage of sports science, exercise physiology, and clinical research related to blood flow restriction training. The search time frame spanned from the inception of each respective database to November 15, 2025, ensuring no relevant studies were omitted due to temporal limitations. The search terms were explicitly defined as follows: (“Blood Flow Restriction Training”, “KAATSU”, “BFR”, “pressure training”, or “occlusion training”) AND (“strength”, “PAP”, “post-activation potentiation”, “upper body”, “lower body”, “fatigue”, or “RPE”) AND (“RCT” or “randomized controlled trial”); these terms were chosen to comprehensively capture studies focusing on BFR training and its effects on muscle strength, PAP, and related outcomes in randomized controlled designs. After initial search and screening, a total of 16 articles were ultimately included in the review, with the detailed screening process documented in the PRISMA flowchart to further improve transparency.

### 2.2 Inclusion and exclusion criteria

To ensure consistency and reduce selection bias, the study inclusion and exclusion criteria were explicitly formulated based on the PICOST principle (Amir-Behghadami and Janati, 2020), with all components clearly defined as follows:

#### 2.2.1 Inclusion criteria

Participants: Healthy individuals (≤35 years old), with no additional age restrictions; all participants were free of chronic diseases, musculoskeletal disorders, or other conditions that could affect exercise performance or the outcomes of BFR training.

Intervention: BFR training with specific pressure parameters (e.g., 60-80% of arterial occlusion pressure) implemented via various modalities (e.g., resistance training, aerobic training); the pressure parameters, training frequency, and duration were clearly reported in each included study.

Control group: Alternative training methods without BFR (e.g., traditional resistance training, traditional aerobic training) or non-training control groups; the control group interventions were matched as closely as possible to the experimental group (except for the absence of BFR) to ensure comparability.

Outcomes: Muscle strength (e.g., 1RM testing for knee extension, bench press), power output (e.g., jump height measured via force plate), functional performance (e.g., 30m sprint time); all outcomes were measured using standardized, validated methods.

Study design: Published randomized controlled trials (RCTs) that explicitly evaluated the effect of BFR training on the lower limb muscle PAP effect; only full-text articles published in peer-reviewed journals were included.

Timeline: Immediate data collected after the completion of each set of exercises; studies were required to report the exact time point of outcome measurement (e.g., 30 seconds, 1 minute post-exercise) to ensure consistency in outcome interpretation.

#### 2.2.2 Exclusion criteria

Non-randomized controlled trial designs: Non-randomized trials, observational studies, systematic reviews, meta-analyses, or case reports; these designs were excluded to minimize selection bias and ensure the reliability of the included evidence.

Irrelevant interventions: Studies that did not involve BFR training, or focused on non-upper/lower limb interventions (e.g., trunk BFR training); studies were only included if they focused on upper or lower limb BFR training.

Population exclusion: Studies involving individuals with chronic diseases (e.g., hypertension, diabetes, cardiovascular disease), pregnant women, or animal models; only studies on healthy human participants were included to avoid confounding factors related to disease or species differences.

Outcome exclusion: Studies lacking quantitative data on muscle strength, power output, fatigue, or PAP outcomes; studies with only qualitative descriptions of outcomes were excluded.

Duplicate publications: Redundant studies with overlapping datasets (e.g., the same study published in multiple journals or as a conference abstract); only the most comprehensive, full-text version of the study was included.

Special handling for inaccessible data: For articles with unavailable full-text or incomplete information (e.g., missing pressure parameters, sample size details), the corresponding authors were contacted via email to request missing materials; non-responsive studies were classified as “pending analysis” and excluded from the final review to ensure data integrity.

Absence of timeline: Studies that failed to provide detailed interval durations (e.g., time between exercise sets, time of outcome measurement post-exercise); these studies were excluded as the timeline is critical for evaluating the PAP effect, which is time-dependent.

### 2.3 Data extraction

**Figure 1:**
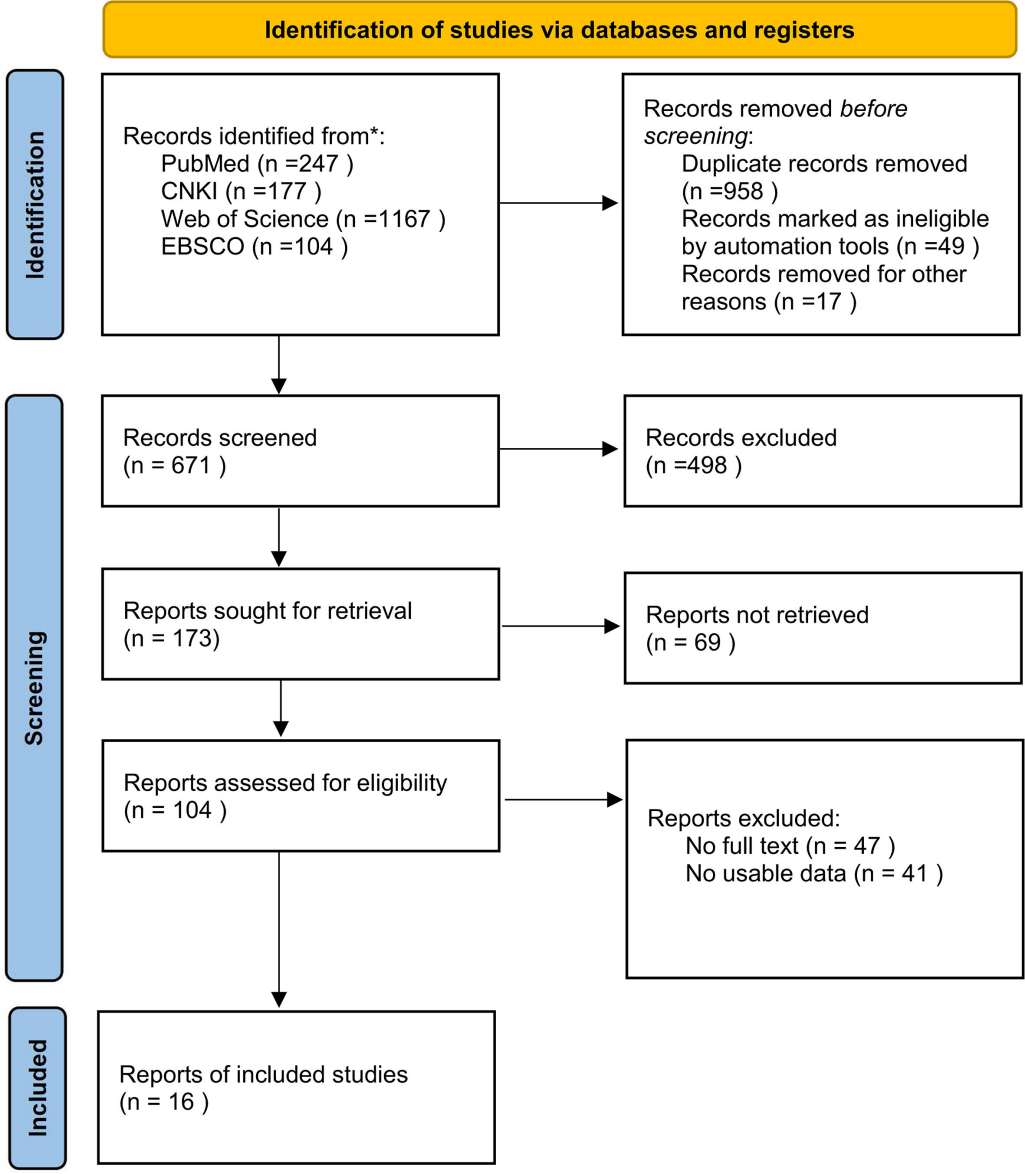
Flow diagram of literature selection.

The retrieved literatures were uniformly imported into NoteExpress 3.9 software to manage and organize the search results. Two independent researchers then conducted a two-stage screening process (title/abstract screening followed by full-text screening) to identify eligible studies, with discrepancies resolved through discussion with a third senior researcher. The PRISMA flowchart (**Figure 1**) elaborates on the detailed procedures of literature screening and inclusion, including the number of studies identified, excluded, and ultimately included at each stage.

Following a rigorous screening process, the two researchers independently extracted relevant information using a self-designed, pre-tested data extraction form to ensure consistency. The extraction form covered the following aspects in detail:

1. General information: Detailed data including the first author, year of publication, country of origin, and journal name to contextualize the included studies.
2. Sample characteristics: Comprehensive information about the research subjects, including their ages (mean ± standard deviation), gender distribution, and the sample sizes of the experimental and control groups; this information was used to assess the homogeneity of the study populations.
3. Characteristics of exercise interventions: The intervention protocols of the experimental group, including detailed parameters such as training modalities (e.g., resistance training with 3 sets of 10 repetitions), training volume (e.g., 3 sessions per week for 8 weeks), pressure intensity (e.g., 70% of arterial occlusion pressure), and occlusion duration (e.g., 2 minutes per set); these factors are crucial for evaluating the nature and intensity of the applied interventions and enabling future replication.
4. Outcome indicators: Quantitative indicators focusing primarily on PAP (e.g., changes in jump height post-activation), interval durations (e.g., time between BFR occlusion and outcome measurement), muscle strength (e.g., 1RM values), and fatigue (e.g., RPE scores); all extracted data were cross-checked by the two researchers to minimize extraction errors.

For data synthesis, the extracted information was thematically organized by intervention type, outcome measure, and study design. Quantitative data were tabulated to facilitate comparison across studies, and discrepancies in study results were noted and discussed to address potential heterogeneity. This synthesis approach ensured that the review findings were comprehensive, transparent, and reproducible.

### 2.4 Statistical analysis

Statistical analyses were performed using Review Manager 5.3 software. The outcome variables reported in the included literature were essentially continuous variables. Considering the variability in measurement methods adopted for different indicators, the standardized mean difference (*SMD*) and 95% confidence interval (95% *CI*) were set as the effect size indices. Subgroup analyses for PAP outcomes were stratified by exercise modality, interval duration, exercise intensity, and pressure intensity. The Cochrane Risk of Bias tool was employed to assess the risk of bias across seven domains: random sequence generation, allocation concealment, blinding of participants and personnel, blinding of outcome assessment, incomplete outcome data, selective reporting, and other sources of bias. Heterogeneity was analyzed via the Q-test, with the predefined significance level set at 0.1. The *I*² statistic ranges from 0% to 100%; a value of *I*² > 50% combined with *P* < 0.1 indicated significant heterogeneity, in which case a random-effects model was selected for the meta-analysis. Otherwise, a fixed-effects model was applied. In addition, Stata 17.0 software was used to conduct sensitivity analyses for verifying the stability of the obtained results. Egger’s test and funnel plot construction were performed to investigate potential publication bias.

## 3 Results

### 3.1 Study characteristics

A total of 16 randomized controlled trials (RCTs) were included in this Meta-analysis, with the study sample comprising 287 participants aged 11 to 35 years old. Detailed characteristics of the included studies are presented in **Table 1**.

**Table 1:** Characteristic of studies included in systematic review and meta-analysis.

Study	Country	Age (years)	N (EG/CG)	Intervention (rest interval)	Plan (BFR intensity)	Outcome extracted
Santiago-Pescador et al. (2022)	Spain	24.8 ± 7.0	15/15	BFR (2min)	25 minute knee exercise (50% AOP)	CMJ↓
Sirieiro et al. (2021)	Brazil	17.3 ± 5.4	16/16	BFR (4min)	1 set of 5 reps at 65% 1RM for half squats(40%AOP)	CMJ↑
Hornikel et al. (2023)	USA	24.8 ± 4.7	13/13	BFR (Immediate)	4 sets of 75%1RM squat 4 sets to failure (80% AOP)	CMJ↓
Bielitzki et al. (2024)	Germany	23.8 ± 3.1	24/24	BFR(8min)	3 sets of 60 s static balance exercise on a BOSU ball(40% AOP)	SJ↑
Doma et al. (2020)	Australia	22.9 ± 5.0	18/18	BFR (Immediate)	3 sets of 8 reps each for leg swings, high knees and hip kicks(130mmHg)	VJ NS
Faller et al. (2023)	USA	20.8 ± 2.4	15/15	BFR (Immediate)	1 set of 6 reps each for hugging walks, side lunges, and buttock bridges(200mmHg)	VJ NS
Zheng et al. (2024)	China	21 ± 2	16/16	BFR (Immediate)	4 groups 15 times 30% 1RM sitting squat(80%AOP)	VJ↑
Miller et al. (2018)	USA	21.8 ± 2.6	20/20	BFR(10min)	3 sets of 20s WVB 3 sets of MVC (160mmHg)	CMJ↑
Yan and Guo (2018)	China	20.3 ± 2.3	20/20	BFR(4min)	5 sets of 50% HRR 2-minute interval runs(150mmHg)	CMJ↑
Cleary and Cook (2020)	USA	20.3 ± 0.9	15/15	BFR(8min)	1 set of 30 times 30% 1RM squats(60%AOP)	VJ NS
Sun and Yang (2023)	China	18.34 ± 1.88	12/12	BFR(5min)	4 setss of 30-15–15–15 times 30%1RM semi-squat(50%AOP)	CMJ↑ SJ↑
Hu (2020)	China	18.3 ± 3.28	12/12	BFR(2min)	2 groups of 30 + 20 30% 1RM squats (bundled pressure 40mmHg, inflation pressure 200mmHg)	CMJ↑
Cornejo-Daza et al. (2024)	Spain	24.5 ± 4.8	20/20	BFR(4min)	3 sets of 8 times 60%1RM full-squat(50%AOP)	CMJ↑
Wei and Xiang (2022)	China	23.6 ± 1.51	27/27	BFR(4min)	2 sets of 10 straight leg jumps+3 sets of 5 consecutive obstacle jumps+5 drop jumps(inflation pressure 160mmHg)	CMJ↑
Lauver et al. (2017)	USA	22.8 ± 2.3	24/24	BFR (Immediate)	4 groups of 30-15-15-15 20%1 RM knee resistance exercises (130% systolic blood pressure)	MVIC↓ RMSvm↑
Leszczynski et al. (2024)	Australia	18-35	20/20	BFR (Immediate)	3 sets of 5min knee extension exercise(80% AOP)	CMJ↑

NS, no statistical significance; ↑ represents a significant increase; ↓ represents a significant decrease; SJ, squat jump; CMJ, counter movement jump; VJ, vertical jump; MVC, maximum autonomous isometric contraction; AOP, arterial occlusion pressure.

### 3.2 Study quality assessment

**Figure 2:**
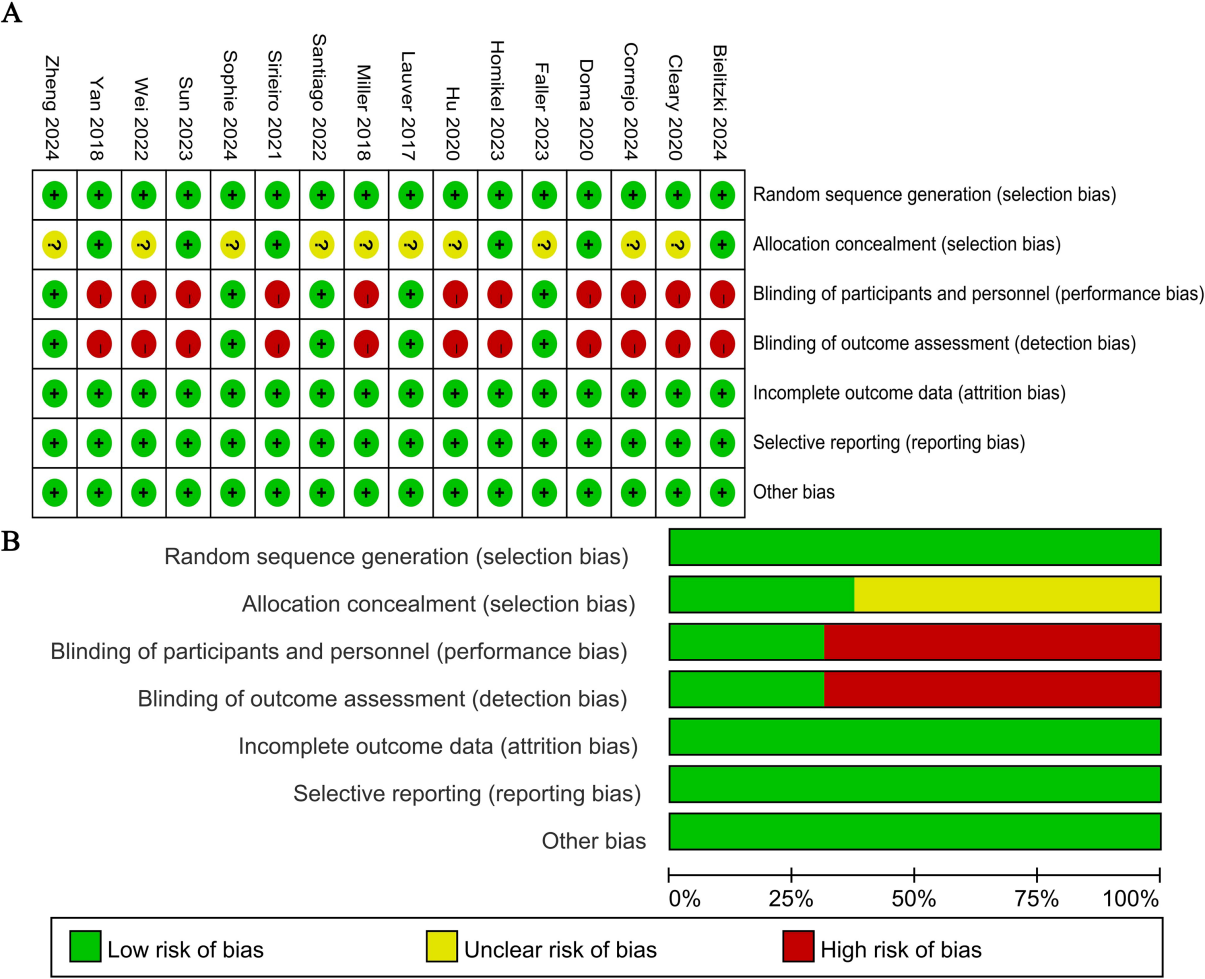
Methodological quality graph and summary of the included studies: **(A)** risk of bias summary; **(B)** risk of bias graph.

Study quality assessment was conducted in accordance with the Cochrane Risk of Bias tool (Page et al., 2021). Seven domains were evaluated using Review Manager 5.3 software, namely random sequence generation, allocation concealment, blinding of participants and personnel, blinding of outcome assessment, incomplete outcome data, selective reporting, and other sources of bias (**Figures 2A, B**). Among the included studies, 10 did not explicitly report whether the randomization procedure was strictly implemented by the allocators. Additionally, 11 studies were identified to have a high risk of bias in blinding, as informed consent was obtained from participants prior to the experiment.

### 3.3 Post-activation potentiation

**Figure 3:**
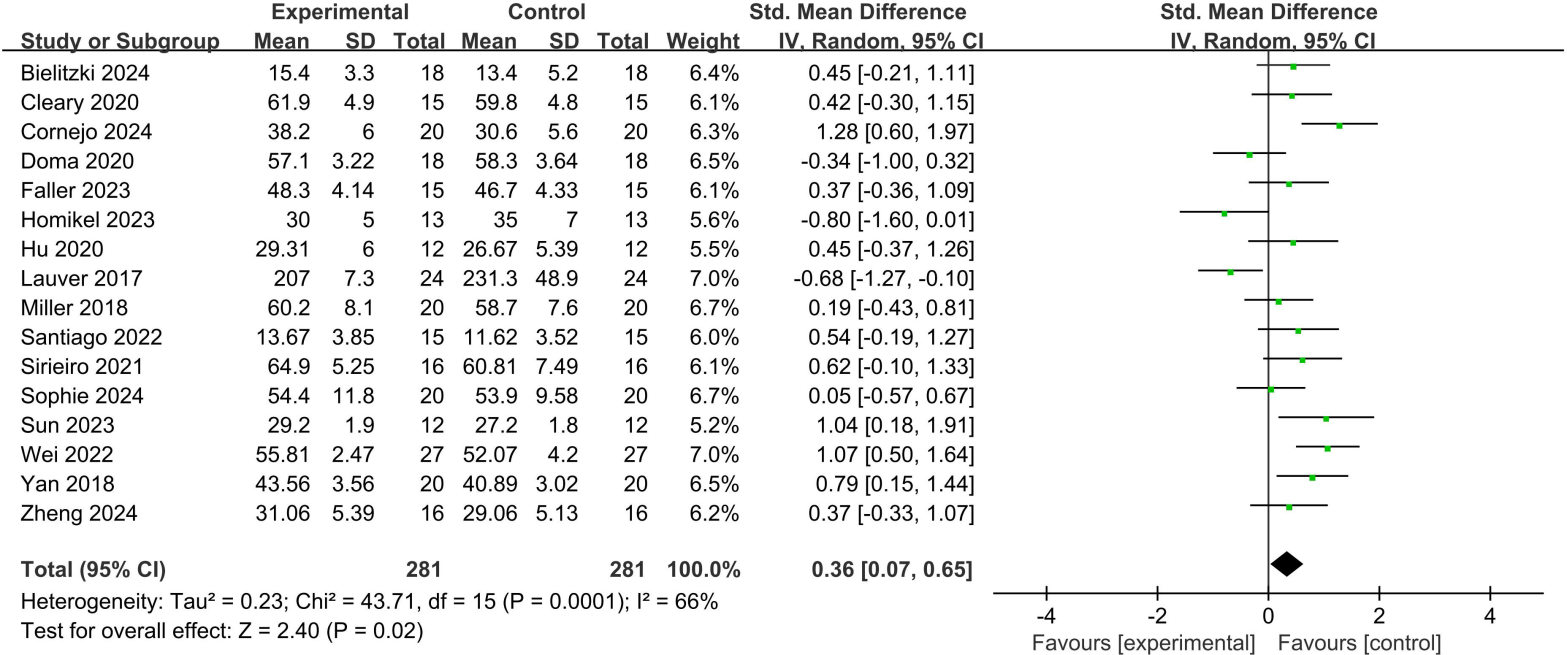
Effect of BFR on PAP.

A total of 16 studies involving 281 participants compared the effect of lower limb BFR exercise on the PAP effect. Heterogeneity testing revealed an *I*² statistic of 66% (> 50%) with a P-value of 0.0001 for the Q-test, indicating significant heterogeneity across the included studies; thus, a random-effects model was selected for the meta-analysis (**Figure 3**). The results showed that the pooled effect size was a standardized mean difference (*SMD*) of 0.36, which was statistically significant (*Z* = 2.40, *P* = 0.02). This finding demonstrated that lower limb BFR exercise could induce the PAP effect compared with the control group.

### 3.4 Subgroup analysis

This study explored the potential sources of heterogeneity through subgroup analyses. The results indicated that rest interval, compressive strength, outcome extracted, exercise mode, and exercise intensity might be the primary factors contributing to the heterogeneity across included studies. Detailed results of each subgroup are presented as follows (**Table 2**):

**Table 2:** Subgroup analysis of lower limb PAP induced by BFR training.

Research features	Subgroup standard	Study (sample)	*SMD*	95% CI	*P*	*I*2 (%)	*P* (Heterogeneity)
Rest interval	Immediate	6(106)	-0.17	-0.57, 0.22	0.39	52	0.06
	2~4 min	6(110)	0.83	0.56, 1.11	<0.0001	0	0.53
	>4 min	4(65)	0.46	0.11, 0.81	0.01	0	0.47
Compressive strength	≤40%AOP	3(52)	0.23	-0.35, 0.81	0.43	55	0.11
	40%~60%AOP	7(129)	0.76	0.46, 1.06	<0.0001	27	0.22
	≥60%AOP	6(100)	-0.05	-0.50, 0.39	0.81	58	0.03
Outcome extracted	MVC	1(24)	-0.68	-1.27,-0.10	0.02	N	N
	CMJ	10(175)	0.53	0.17, 0.89	0.004	63	0.004
	SJ	1(18)	0.45	-0.21, 1.11	0.33	N	N
	VJ	4(64)	0.18	-0.19, 0.55	0.33	11	0.34
Exercise mode	Squat	7(104)	0.49	0.02, 0.97	0.04	64	0.01
	Combined Exercise	5(98)	0.36	-0.11, 0.64	0.13	62	0.03
	Knee Extension	3(59)	-0.06	-0.75, 0.64	0.87	71	0.03
	Running	1(20)	0.79	0.15, 1.44	0.06	N	N
Exercise intensity	≤30% 1RM	5(79)	0.17	-0.15, 0.49	0.31	70	0.10
	40%~70% 1RM	2(36)	0.99	0.48, 1.51	0.0002	37	0.21
	Exhaustive exercise	1(13)	-0.80	-1.60, 0.01	0.05	N	N
	Combined Intensity	8(153)	0.41	0.18, 0.64	0.0005	48	0.06

N, no applicable.

For the rest interval subgroup analysis, the heterogeneity of all subgroups with different rest intervals was lower than that of the overall pooled effect (*I*² = 66%). Specifically, the subgroup with Immediate rest interval showed a heterogeneity of 52%, while the heterogeneity of the other two subgroups was reduced to 0%. The effect size analysis demonstrated that the subgroup with a 2–4 min rest interval yielded the highest effect size with statistical significance (*SMD* = 0.83, P<0.0001), suggesting that this rest interval duration is more conducive to the induction of the PAP effect in lower limbs.

In the compressive strength subgroup, the studies in the ≤40% AOP and ≥60% AOP subgroups exhibited high heterogeneity (*I*² = 55% and 58%, respectively), whereas the 40%–60% AOP subgroup showed relatively low heterogeneity (*I*² = 27%). The effect size results revealed that the 40%–60% AOP subgroup had the largest effect size with statistical significance (*SMD* = 0.76, *P* < 0.0001), indicating that this range of compressive strength is more likely to induce the PAP effect.

Within the outcome extracted subgroup, the subgroup using countermovement jump (CMJ) height as the outcome measure presented the highest heterogeneity (*I*² = 66%), which was consistent with that of the overall pooled effect. Meanwhile, this subgroup also had the largest effect size with statistical significance (*SMD* = 0.53, *P* = 0.004). These findings demonstrated that BFR training could significantly improve the CMJ height of participants, i.e., exerting a positive effect on lower limb explosive power.

In the exercise mode subgroup, the heterogeneity of each subcategory did not decrease significantly compared with that of the overall pooled effect (*I*² = 66%). Among them, the Knee Extension subgroup showed a further increase in heterogeneity (*I*² = 77%), implying that the included studies in this subgroup had substantial methodological or population characteristic differences. The Squat subgroup had a heterogeneity of 62% and the largest effect size with statistical significance (*SMD* = 0.49, *P* = 0.04), indicating that squat exercises combined with BFR are more likely to induce the PAP effect.

For the exercise intensity subgroup, the ≤30% 1RM subgroup exhibited the strongest heterogeneity (*I*² = 70%). In contrast, the 40%–70% 1RM subgroup showed relatively low heterogeneity and the largest effect size with statistical significance (*SMD* = 0.99, *P* = 0.0002). The results suggested that compared with the exercise intensities of ≤30% 1RM and >70% 1RM, BFR training at an intensity of 40%–70% 1RM is more favorable for PAP induction.

### 3.5 Sensitivity analysis

Sensitivity analysis was performed using the leave-one-out method to evaluate the impact of individual included studies on the overall heterogeneity of the meta-analysis.

As shown in **Table 3**, the pooled effect size of BFR training on the PAP effect was [*SMD* = 0.36, 95% *CI* (0.07, 0.65), *P* = 0.02]. After sequentially excluding each individual study, the pooled *SMD* fluctuated within the range of 0.30 to 0.44, and the heterogeneity index I² ranged from 54% to 68%. No single study exerted a significant impact on the pooled effect size of the meta-analysis, indicating that the results of this study have good stability.

**Table 3:** Combined effects of jumping ability after excluding individual studies.

Study	*SMD*	95% *CI*	*P* (merge effect)	*I^2^* (%)
Bielitzki et al. (2024)	0.35	0.04, 0.67	0.04	68
Cleary and Cook (2020)	0.36	0.04, 0.67	0.04	68
Cornejo-Daza et al. (2024)	0.30	0.01, 0.58	0.06	61
Doma et al. (2020)	0.40	0.11, 0.70	0.01	64
Faller et al. (2023)	0.36	0.05, 0.67	0.02	68
Hornikel et al. (2023)	0.43	0.15, 0.70	0.003	61
Hu (2020)	0.35	0.04, 0.68	0.03	68
Lauver et al. (2017)	0.44	0.17, 0.70	0.001	54
Miller et al. (2018)	0.37	0.06, 0.69	0.02	68
Santiago-Pescador et al. (2022)	0.35	0.04, 0.66	0.03	68
Sirieiro et al. (2021)	0.34	0.03, 0.65	0.02	68
Leszczynski et al. (2024)	0.38	0.07, 0.69	0.03	67
Sun and Yang (2023)	0.32	0.02, 0.62	0.02	66
Wei and Xiang (2022)	0.30	0.01, 0.60	0.04	62
Yan and Guo (2018)	0.33	0.02, 0.64	0.04	66
Zheng et al. (2024)	0.36	0.05, 0.67	0.02	68
Overall	0.36	0.07, 0.65	0.02	66

### 3.6 Publication bias

**Figure 4:**
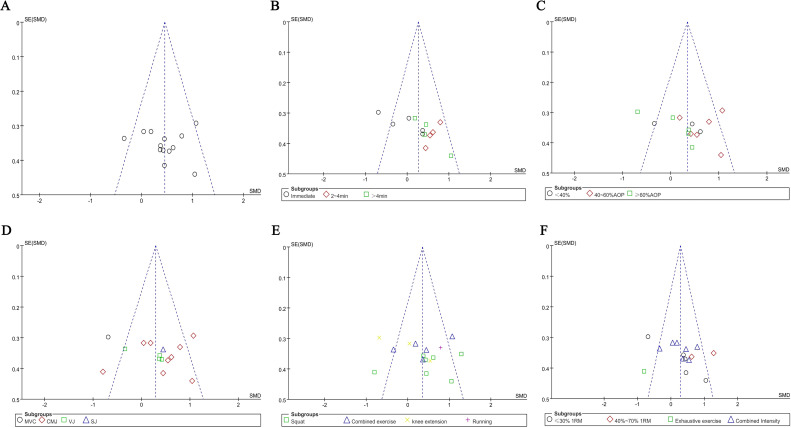
Funnel plots of PAP: **(A)** combine funnel chart; **(B)** rest interval; **(C)** compressive strength; **(D)** outcome extracted; **(E)** exercise mode; **(F)** exercise intensity.

Funnel plots were separately generated for each subgroup to assess potential publication bias. As shown in **Figure 4**, the funnel plots exhibited a roughly symmetrical distribution. Egger’s test was further performed on the aforementioned funnel plots, and the results indicated that the P-values of all subgroups were greater than 0.05, demonstrating the absence of significant publication bias among the included studies.

## 4 Discussion

### 4.1 The effect of BFR on PAP

The present meta-analysis synthesized data from 16 studies involving 281 participants to systematically evaluate the effect of lower limb BFR exercise on PAP. The results revealed that lower limb BFR exercise could induce significant PAP compared with the control group, as indicated by a pooled effect size of *SMD* = 0.36 (*Z* = 2.40, *P* = 0.02). This finding contrasts with Wang et al. (2023), who found no significant PAP induction in their sample of elderly participants—a discrepancy likely attributed to age-related declines in neuromuscular excitability and metabolic stress responsiveness, which we will elaborate on further in subsequent sections. This finding provides robust evidence for the application of BFR exercise in enhancing acute neuromuscular performance, which is of great significance for optimizing training strategies in athletic populations and improving functional recovery in clinical rehabilitation settings.This variation highlights the critical role of intervention standardization—an issue that has plagued the BFR-PAP literature and represents a key gap we aim to address.

The potential mechanisms underlying the induction of PAP by lower limb BFR exercise can be elaborated from two core aspects: the cumulative effect of metabolic stress and the adaptive regulation of neuromuscular function. Firstly, BFR exercise induces local hypoxia and accumulation of metabolic by-products (e.g., lactate, hydrogen ions, and adenosine) in the working muscles by restricting venous return while maintaining arterial perfusion (Rivera et al., 2022). These metabolic intermediates can activate group III/IV afferent nerve fibers, which in turn modulate the excitability of the spinal motor neuron pool and enhance the recruitment of high-threshold motor units (HTMUs) (Pignanelli et al., 2021). HTMUs are predominantly composed of type II muscle fibers with high contractile velocity and force-generating capacity, and their enhanced recruitment is a key physiological basis for the occurrence of PAP (Zheng et al., 2022).

Notably, while most included studies attributed PAP to HTMU recruitment, two studies (Doma et al., 2020; Faller et al., 2023) failed to measure motor unit activation directly, relying instead on indirect outcome measures (e.g., jump height), which introduces uncertainty into the mechanistic interpretation—a critical gap in the current literature that limits our ability to confirm causal links between BFR and PAP. Compared with conventional non-BFR exercise, BFR exercise can achieve the activation of HTMUs at a lower exercise intensity (30%-50% of 1-repetition maximum, 1RM), thereby reducing the mechanical load on the musculoskeletal system while inducing effective PAP (Zeng et al., 2025).

Secondly, BFR exercise may regulate the phosphorylation level of myosin regulatory light chains (RLCs) to promote PAP. The phosphorylation of RLCs can increase the sensitivity of actin-myosin cross-bridge formation to calcium ions, thereby enhancing muscle contraction efficiency (Zhang et al., 2022). Studies have shown that metabolic stress induced by BFR can up-regulate the activity of myosin light chain kinase (MLCK), which is the key enzyme mediating RLCs phosphorylation (Xia et al., 2019). However, inconsistencies exist in the literature regarding this mechanism: while Liu et al. (2024) (Liu et al., 2024) found no significant changes, possibly due to differences in exercise intensity (30% vs. 60% 1RM) and pressure parameters (50% vs. 70% AOP). This inconsistency underscores the need for standardized intervention protocols to resolve conflicting mechanistic findings. In addition, local hypoxia caused by BFR may also promote the release of calcium ions from the sarcoplasmic reticulum, further enhancing the interaction between actin and myosin and strengthening the potentiation effect. This dual regulation of metabolic stress on neuromuscular recruitment and cross-bridge formation may be the primary reason why lower limb BFR exercise can effectively induce PAP.

### 4.2 Moderating factors of PAP in lower limbs

Notably, the heterogeneity test in this meta-analysis showed significant heterogeneity among the included studies (*I*² = 66%, *P* = 0.0001), which is consistent with the characteristics of most exercise intervention meta-analyses. To further clarify the potential sources of heterogeneity and optimize the clinical application of BFR exercise for PAP induction, we conducted subgroup analyses based on key intervention and detection parameters. The results summarized above confirmed that rest interval, compressive strength, outcome extracted, exercise mode, and exercise intensity are the main factors contributing to the inter-study heterogeneity. Our critical analysis of these moderators reveals that no single parameter acts in isolation—rather, their interaction determines the magnitude of PAP, a finding that has been overlooked in most previous reviews, which tend to focus on individual factors in isolation. This perspective positions our work as a novel contribution to the field, emphasizing the need for personalized, parameter-matched BFR protocols rather than one-size-fits-all approaches.

#### 4.2.1 Rest interval

For the rest interval subgroup, the heterogeneity of all subgroups was lower than that of the overall pooled effect (*I*² = 66%). Specifically, the heterogeneity was 52% in the immediate rest interval group, and reduced to 0% in the other two groups, indicating that standardizing the rest interval can effectively reduce the inconsistency among studies. Regarding the effect size, the 2~4 min rest interval group showed the highest effect size with statistical significance (*SMD* = 0.83, *P*<0.0001), suggesting that this rest interval is more conducive to the generation of lower limb PAP. The underlying mechanism may be related to the balance between metabolic stress accumulation and partial recovery of neuromuscular function. Immediate testing may lead to insufficient clearance of metabolic by-products (e.g., lactate), which may inhibit the excitability of motor neurons; while excessively long rest intervals (longer than 4 min) may reduce the residual metabolic stress and the excitability of high-threshold motor units (HTMUs), thereby weakening the PAP effect (Hughes and Patterson, 2019). In contrast, a 2~4 min rest interval allows for moderate clearance of metabolic inhibitors while maintaining the enhanced excitability of the neuromuscular system induced by BFR exercise, which optimizes the PAP response (Fan et al., 2023). A key gap identified here is that no included study measured metabolic markers (e.g., lactate, adenosine) alongside PAP outcomes, making it impossible to directly link rest interval-induced changes in metabolic stress to PAP magnitude—this is a critical direction for future research.

#### 4.2.2 Compressive strength

In the compressive strength subgroup, the ≤ 40%AOP and ≥ 60%AOP groups exhibited strong heterogeneity (*I*² = 55% and 58%, respectively), while the 40%~60%AOP group had relatively low heterogeneity (*I*² = 27%). The effect size results showed that the 40%~60%AOP group had the largest effect size with statistical significance (*SMD* = 0.76, *P* < 0.0001), indicating that this pressure range is more likely to induce PAP. This phenomenon can be explained by the “metabolic stress threshold theory”: when the compressive strength is lower than 40%AOP, the restriction on venous return is insufficient, resulting in inadequate accumulation of metabolic by-products and weak activation of afferent nerve fibers, thus failing to induce effective PAP (Lau et al., 2023). When the compressive strength is higher than 60%AOP, it may not only restrict venous return but also affect arterial perfusion, leading to severe muscle hypoxia and cell damage, which counteracts the potentiation effect and increases the heterogeneity due to individual differences in tolerance to high pressure (Doma et al., 2020). In contrast, the 40%~60%AOP range can precisely balance venous restriction and arterial perfusion, maximizing metabolic stress without causing excessive ischemia, thereby inducing a stable and strong PAP effect (Herrera and Osorio-Fuentealba, 2024). From our perspective, the 40%~60%AOP range represents an optimal “window” for PAP induction across diverse populations, which has important practical implications for standardizing BFR protocols in both athletic and clinical settings.

#### 4.2.3 Outcome extractede

For the outcome extracted subgroup, the group with CMJ height as the test index had the highest heterogeneity (*I*² = 66%), consistent with the overall pooled effect heterogeneity. Meanwhile, this group had the largest effect size with statistical significance (*SMD* = 0.53, *P* = 0.004), indicating that BFR training can significantly improve participants’ CMJ height, i.e., have a positive impact on lower limb explosive power. The high heterogeneity in the CMJ group may be attributed to the differences in CMJ testing protocols (e.g., whether arm swing is allowed, take-off speed) and the training level of participants among different studies (Zhou et al., 2024). The superior effect of BFR exercise on CMJ height may be related to the specific enhancement of type II muscle fiber function. CMJ is a typical explosive movement dominated by type II muscle fibers, and BFR exercise-induced metabolic stress can specifically activate HTMUs (mainly composed of type II fibers) and improve the efficiency of actin-myosin cross-bridge formation, thereby significantly enhancing explosive power output (Chen et al., 2025). In contrast, other test indicators (e.g., isometric torque) may be more dependent on muscle endurance or static strength, resulting in relatively smaller effect sizes (Wortman et al., 2021).

#### 4.2.4 Exercise mode

In the exercise mode subgroup, the heterogeneity of each group did not decrease significantly compared with the overall pooled effect (*I*² = 66%), and the heterogeneity of the knee extension group further increased (*I*² = 77%), suggesting large methodological or population characteristic differences among the included studies in this group. The squat group had a heterogeneity of 62% and the largest effect size with statistical significance (*SMD* = 0.49, *P* = 0.04), indicating that squat exercise combined with BFR is more likely to produce the PAP effect. The higher heterogeneity in the knee extension group may be due to differences in the range of motion, angular velocity, and fixation methods of the knee joint during testing among studies, which affect the activation pattern of lower limb muscles (Jack et al., 2023). In contrast, squat is a multi-joint compound movement that can activate more muscle groups (e.g., quadriceps, glutes, hamstrings) simultaneously, leading to more comprehensive metabolic stress accumulation and neuromuscular activation (Yuksel et al., 2025). This comprehensive activation can induce a more significant PAP effect by coordinating the function of multiple muscle groups, which explains the larger effect size in the squat group (Kong et al., 2022).

#### 4.2.5 Exercise intensity

In the exercise intensity subgroup, the ≤30% 1RM group had the strongest heterogeneity (*I*² = 70%), while the 40%~70% 1RM group had relatively low heterogeneity and the largest effect size with statistical significance (*SMD* = 0.99, P = 0.0002). The results suggest that compared with ≤30% 1RM and >70% 1RM exercise intensities, BFR training at 40%~70% 1RM is more conducive to PAP induction. A critical comparison across intensity groups reveals a U-shaped relationship between exercise intensity and PAP magnitude: too low (≤30% 1RM) leads to insufficient mechanical stimulation, while too high (>70% 1RM) causes acute fatigue that inhibits PAP (Zhou et al., 2026).The mechanism may be related to the balance between mechanical load and metabolic stress. When the exercise intensity is lower than 30% 1RM, the mechanical stimulation is insufficient to activate HTMUs, and even with BFR, the metabolic stress accumulation is limited, resulting in weak PAP and high heterogeneity due to individual differences in neuromuscular sensitivity (Blazevich and Babault, 2019). When the exercise intensity is higher than 70% 1RM, the excessive mechanical load may cause acute muscle fatigue, which inhibits the PAP effect, and the combination of high intensity and BFR may increase the risk of muscle damage, leading to inconsistent responses among individuals (Gago et al., 2020). The 40%~70% 1RM intensity range can combine appropriate mechanical stimulation with BFR-induced metabolic stress, maximizing the activation of HTMUs and the phosphorylation of myosin RLCs without causing excessive fatigue, thus achieving the optimal PAP effect (Hodgson et al., 2005).

The clinical and practical implications of this study are notable. For athletes, lower limb BFR exercise can be used as a pre-competition activation strategy. Compared with traditional high-intensity pre-activation exercises, BFR exercise with lower intensity can reduce the risk of muscle fatigue and injury while inducing effective PAP, thereby improving competitive performance (Karanasios et al., 2022). For patients with musculoskeletal diseases (e.g., knee osteoarthritis) or the elderly with decreased muscle function, BFR exercise can induce PAP with a lower mechanical load, which is conducive to improving their motor function and balance ability and reducing the risk of falls (Hughes et al., 2017).

### 4.3 Study limitations

This study also has certain limitations. Firstly, the number of included studies and participants is relatively limited, which may affect the stability and generalizability of the results. Secondly, due to the lack of detailed data in some included studies, we were unable to conduct subgroup analyses based on BFR pressure intensity, exercise mode, and participant training level, which makes it impossible to clarify the key factors affecting the PAP effect of BFR exercise. Thirdly, most of the included studies are short-term intervention studies, and the long-term adaptation effect of BFR exercise on PAP and its potential adverse reactions (e.g., muscle damage, thrombosis) need to be further verified by high-quality long-term follow-up studies. Critically, these limitations highlight key gaps in the current literature: the lack of standardized BFR protocols, insufficient long-term data, and limited focus on vulnerable populations (e.g., elderly, clinical patients). These gaps are not trivial—they hinder the translation of BFR-PAP research into clinical and athletic practice, which is a core challenge our work aims to address. From our perspective, future studies should prioritize three key areas to advance the field: (1) standardize BFR intervention and outcome measurement protocols to reduce inter-study heterogeneity, (2) expand sample sizes and include diverse populations (e.g., elderly, clinical patients) to improve generalizability, and (3) conduct long-term follow-up studies to evaluate safety and long-term efficacy. Additionally, mechanistic studies should integrate metabolic and neuromuscular measurements (e.g., lactate levels, motor unit activation) to confirm causal links between BFR parameters and PAP. This direction aligns with our core conclusion that personalized, standardized BFR protocols are essential for maximizing PAP effects and ensuring safe application.

In addition, long-term intervention studies are needed to evaluate the safety and long-term efficacy of BFR exercise, and to explore the molecular mechanisms underlying the regulation of PAP by BFR exercise at the cellular and molecular levels.

## 5 Conclusion

Lower limb BFR training can effectively induce PAP. For lower limb BFR interventions targeting PAP induction, squat exercises with an exercise intensity of 40%–70% 1RM, compressive strength of 40%–60% AOP, and an inter-set rest interval of 2–4 min yield the optimal potentiation effect.

## Data Availability

The original contributions presented in the study are included in the article/supplementary material, further inquiries can be directed to the corresponding author/s.
